# Anti-HIV Activities and Mechanism of 12-*O*-Tricosanoylphorbol-20-acetate, a Novel Phorbol Ester from *Ostodes katharinae*

**DOI:** 10.3390/molecules22091498

**Published:** 2017-09-08

**Authors:** Huan Chen, Rong Zhang, Rong-Hua Luo, Liu-Meng Yang, Rui-Rui Wang, Xiao-Jiang Hao, Yong-Tang Zheng

**Affiliations:** 1 Key Laboratory of Bioactive Peptides of Yunnan Province/Key Laboratory of Animal Models and Human Disease Mechanisms of the Chinese Academy of Sciences, Kunming Institute of Zoology, Chinese Academy of Sciences, Kunming 650223, Yunnan, China; qingxing1214@163.com (H.C.); luorh@mail.kiz.ac.cn (R.-H.L.); lmyang@mail.kiz.ac.cn (L.-M.Y.); wangrr1980@163.com (R.-R.W.); 2 State Key Laboratory of Phytochemistry and Plant Resources in West China, Kunming Institute of Botany, Chinese Academy of Sciences, Kunming 650201, Yunnan, China; zr28875@yangzijiang.com; 3 Kunming College of Life Science, University of Chinese Academy of Sciences, Kunming 650204, Yunnan, China; 4 KIZ-SU Joint Laboratory of Animal Models and Drug Development, College of Pharmaceutical Sciences, Soochow University, Suzhou 215006, Jiangsu, China

**Keywords:** antiviral agent, 12-*O*-tricosanoylphorbol-20-acetate, HIV, Vif, APOBEC3G, phorbol ester

## Abstract

APOBEC3G is a member of the human cytidine deaminase family that restricts Vif-deficient viruses by being packaged with progeny virions and inducing the G to A mutation during the synthesis of HIV-1 viral DNA when the progeny virus infects new cells. HIV-1 Vif protein resists the activity of A3G by mediating A3G degradation. Phorbol esters are plant-derived organic compounds belonging to the tigliane family of diterpenes and could activate the PKC pathway. In this study, we identified an inhibitor 12-*O*-tricosanoylphorbol-20-acetate (hop-8), a novel ester of phorbol which was isolated from *Ostodes katharinae* of the family Euphorbiaceae, that inhibited the replication of wild-type HIV-1 and HIV-2 strains and drug-resistant strains broadly both in C8166 cells and PBMCs with low cytotoxicity and the EC_50_ values ranged from 0.106 μM to 7.987 μM. One of the main mechanisms of hop-8 is to stimulate A3G expressing in HIV-1 producing cells and upregulate the A3G level in progeny virions, which results in reducing the infectivity of the progeny virus. This novel mechanism of hop-8 inhibition of HIV replication might represents a promising approach for developing new therapeutics for HIV infection.

## 1. Introduction

Human immunodeficiency virus (HIV) is the causative agent of acquired immunodeficiency syndrome (AIDS). The highly active antiretroviral therapy (HAART) reduces the plasma viral load and prolongs the lifespan of people with HIV [1]. However, the high mutation rate of HIV genomic RNA, toxicity, side effects of the drugs, and poor patient adherence are major factors that result in treatment failure [2,3,4,5]. Therefore, research on new potential targets and novel drugs for HIV treatment is critically important.

The replication of HIV-1 in host cells cannot be completed without utilizing host cell receptors and factors, such as CD4, CCR5, CXCR4, cyclophilin A, and LEDGF/p75 [6]. Conversely, host cells also express restriction factors such as TRIM5α, tetherin, and the cytidine deaminase protein apolipoprotein B mRNA editing enzyme, catalytic polypeptide-like 3 (APOBEC3) to suppress viral replication. Some HIV auxiliary proteins neutralize the functions of these restriction factors [7,8,9,10]. Viral–host protein interactions have become novel targets for the design and development of potential anti-HIV drugs. The restriction factor APOBEC3G (A3G) belongs to the cell cytidine deaminase family, which exhibits potent antiviral activity against retrotransposition and retroviruses including HIV and hepatitis B virus (HBV) [11,12,13,14,15,16]. A3G restricts HIV replication very efficiently by incorporating into the newly assembled virions and inducing G→A mutations in the minus strand of the newly synthesized viral cDNA [17,18,19]. However, the virus infective factor (Vif), which plays very important roles in HIV infection, inhibits A3G through proteasome-mediated degradation and degradation-independent mechanisms and allows the virus to infect new cells [15,16,20,21,22,23,24]. Vif exploits the Elongin C/B (ELOC/B), Cullin 5 (CUL5), and CBF-β complex to induce A3G ubiquitination and proteasome degradation [23,25,26,27,28,29]. Following studies of the A3G-Vif-CBF-β-CUL5-ELOB-ELOC complex, inhibitors that target the Vif–A3G interaction have been designed to protect A3G from Vif-mediated degradation [30,31,32,33,34,35]. Meanwhile, regulation of A3G expression has been investigated for several years. The expression of A3G is strongly induced by the type I interferon (IFN) mediated pathway in liver cells and macrophages [36,37]. Given A3G’s highly efficient interruption of HIV-1 replication, upregulating A3G expression provides a potential method for restricting wild-type HIV-1 replication.

**Figure 1:**
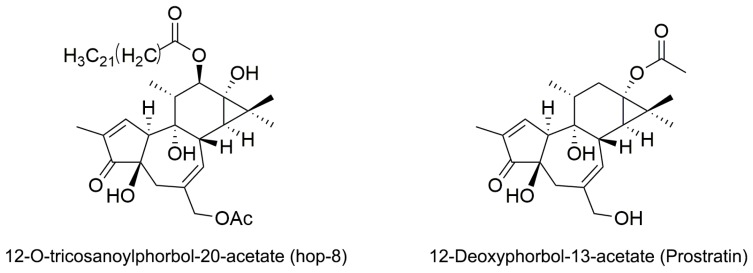
Structure of 12-*O*-tricosanoylphorbol-20-acetate (hop-8) and 12-Deoxyphorbol-13-acetate (prostratin).

Phorbol is a natural, plant-derived organic compound that belongs to the tigliane family of diterpenes. Phorbol esters are esterified derivatives of phorbol. Phorbol myristate acetate (PMA), the most common phorbol ester, strongly induces A3G expression in proliferating H9 cells through the protein kinase C (PKC) α/βI/MEK/ERK pathway [38,39]. However, the tumor-promoting activity of PMA hinders its therapeutic use [40]. Various phorbol esters have been studied as anti-HIV inhibitors and HIV-1 latency activators, but many of them induce tumors [41,42,43]. The discovery of non-tumorigenic phorbol esters prostratin (12-deoxyphorbol-13-acetate) and DPP (12-deoxyphorbol-13-phenylacetate) that inhibit HIV replication in vitro opens up the possibilities for their clinical use [43,44,45]. Prostratin inhibits HIV-1 infections by down regulating HIV-1 cellular receptors and activate HIV-1 latency through the activation of PKC pathway [46]. In this study, we identified a novel ester of phorbol 12-*O*-tricosanoylphorbol-20-acetate (hop-8) (Figure 1) which was isolated from *Ostodes katharinae* of the family Euphorbiaceae, that could strongly enhance A3G expression in cells, reduce the infectivity of the progeny virus, and inhibit laboratory-adapted strains, drug-resistant strains, and clinical isolates of HIV-1 and HIV-2 broadly with low cytotoxicity.

## 2. Results

### 2.1. The Structure Elucidation of Hop-8

Hop-8 was obtained as colorless oil, ${\left[\alpha \right]}_{D}^{16}$ +20.02 (*c* 0.12, CH_3_Cl). Its molecular formula was determined as C_45_H_74_O_8_ based on the HREIMS *m/z* 742.5022 (calcd. for 742.5784) as well as ESIMS *m*/*z* 765 [M + Na]^+^, indicating nine degrees of unsaturation. The structure of hop-8 could be identified to be phorbol ester by its typical ^1^H-NMR signals of phorbol-type diterpenes as follows: δ_H_ 7.59 (1H, s, H-1), 5.70 (1H, d, *J* = 4.5 Hz, H-7), 5.41 (1H, d, *J* = 10.0 Hz, H-12), 4.47(1H, d, *J* = 12.5 Hz, H-20), 4.42 (1H, d, *J* = 12.5 Hz, H-20), 2.52 (1H, d, *J* = 12.0 Hz, H-5), 2.42 (1H, d, *J* = 12.0 Hz, H-5), 1.05 (1H, d, *J* = 5.1 Hz, H-14), 0.85 (3H, d, *J* = 5.7 Hz, H_3_-18), and 1.75 (3H, s, H_3_-19). However, there is an additional fatty acid moiety as well as an acetic acid moiety in hop-8 compared with phorbol. The absorption bands of its IR spectrum also indicated the presence of ester groups (1748, 1730, and 1261 cm^−1^). Considering its molecular formula, fatty acyl could be a tricosanoyl, which further demonstrated by the fragments of EI^+^ MS spectrum *m*/*z*: 403 (13), 387 (15), 310 (100), and 282 (30). Compared ^1^H-NMR data of hop-8 with phorbol [47], markedly downfield shifts of ^1^H-NMR signals of H-12 and H-20 in hop-8 suggested two acyl groups linked in 12-OH and 20-OH, respectively. A cross peak between the signal of carbonyl carbon [δ_C_ 173.5 (C-1′)] and H-12 [δ_H_ 5.41 (1H, d, *J* = 10.0 Hz, H-12)] indicated the tricosanoyl linked at 12-OH, while the acetyl group of hop-8 then linked at 20-OH. The relative configuration was established by its NOESY correction spectrum of NMR. The structure of hop-8 was thus identified as 12-*O*-tricosanoylphorbol-20-acetate.

### 2.2. Hop-8 Significantly Inhibited the Replication of Lab-Adaped HIV-1 and HIV-2, Clinical Isolate Strains, and Drug-Resistant Strains with Low Cytotoxicity

**Figure 2:**
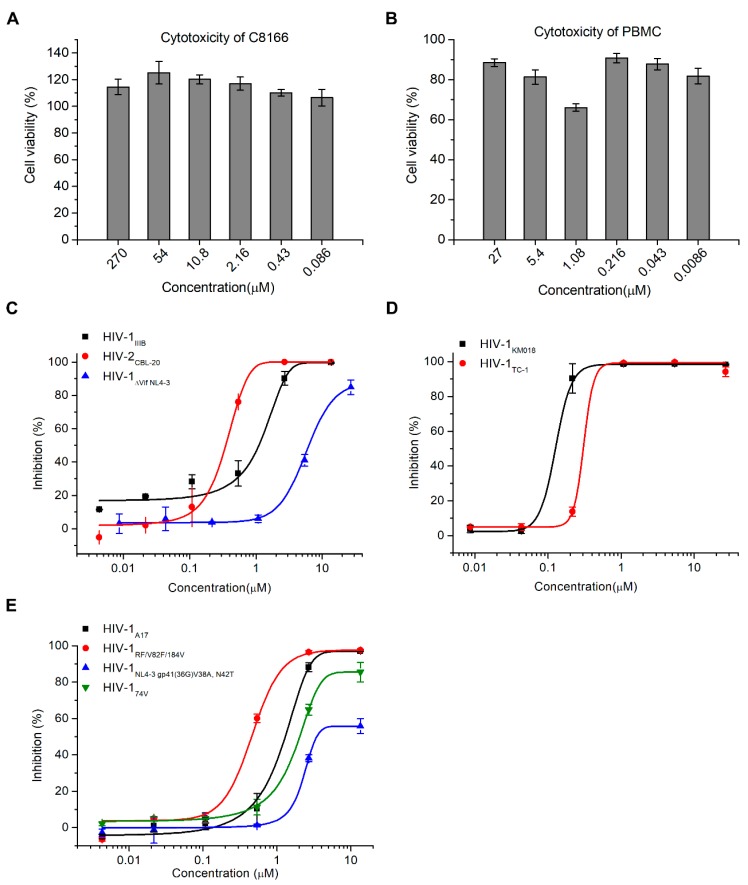
Hop-8 inhibited HIV-1 and HIV-2 wild-type, clinical isolates and resistant strains with low cytotoxicity. (**A**) The cytotoxicity of hop-8 in C8166; (**B**) The cytotoxicity of hop-8 in peripheral blood mononuclear cells (PBMCs); (**C**) The antiviral activities of hop-8 against the clinical isolates HIV-1_KM018_, and HIV-1_TC-1_ were measured in PBMCs; (**D**) The antiviral activities of hop-8 against wild-type HIV-1_IIIB_, HIV-2_CBL-20_, and HIV-1_ΔVif NL4-3_ were measured in the C8166 cell line; (**E**) Antiviral activities of hop-8 against the HIV-1 drug resistant strains HIV-1_A17_, HIV-1_NL4-3 gp41 (36G) V38A, N42T_, HIV-1_RF/V28F/184V_, and HIV-1_74V_. The levels of p24 in the cell culture supernatant were measured by ELISA. Each data point represents the mean percent inhibition (relative to the positive control) ± standard deviation (bars), n ≥ 2. Data were analyzed with Origin 8.5 (OriginLab, MA, USA).

To evaluate the antiviral activity of hop-8, the anti-HIV activity assay and cytotoxicity assay were performed. Prostratin was used as positive control. No cytotoxicity was observed at a concentration of 270 μM of the compound in T cell line C8166 cells and 27 μM in peripheral blood mononuclear cells (PBMCs) (Figure 2A,B). The antiviral activity of hop-8 was evaluated in PBMCs and C8166 cells. Hop-8 inhibited the replication of the HIV-1 clinical isolates KM018 (R5 tropism) and TC-1 (X4 tropism) in PBMCs efficiently, with EC_50_ values 0.106 ± 0.003 and 0.390 ± 0.038 μM, respectively (Table 1, Figure 2B). Hop-8 also showed antiviral activity against HIV-1_IIIB_ and HIV-2_CBL-20_ in C8166 cells with EC_50_ values 0.873 ± 0.005 and 0.255 ± 0.023 μM, respectively. However, hop-8 inhibited the *vif* deficient strain (ΔVif NL4-3) less efficiently with an EC_50_ 7.987 ± 0.481 μg/mL, and compared to the EC_50_ of HIV-1_IIIB_, it represented a fold change of 9.1 (Table 1, Figure 2C). The antiviral activity of hop-8 against drug-resistant strains of HIV-1 was also measured in the C8166 cell line. The HIV-1 strains NL4-3 gp41 (36G) V38A, N42T (fusion inhibitor resistant strain), A17 (non-nucleoside reverse transcriptase inhibitor resistant strain), RF/V82F/184V (protease inhibitor resistant strain), or 74V (nucleoside reverse transcriptase inhibitor resistant strain) were used to infect C8166 cells. Hop-8 showed good antiviral activity against drug-resistant strains. EC_50_ values ranged from 0.396 to 6.915 μM (Table 1 and Figure 2D). Prostratin was used as a control (Table 1). The antiviral activity of hop-8 is better than prostratin. These results indicated that hop-8 is a broad-spectrum inhibitor of HIV that efficiently inhibits lab-adapted, drug-resistant, clinically isolated strains of HIV-1 in different subtypes as well as HIV-2 with low cytotoxicity.

### 2.3. Hop-8 Restored A3G Levels in Cells Undergoing Vif-Mediated A3G Degradation

**Figure 3:**
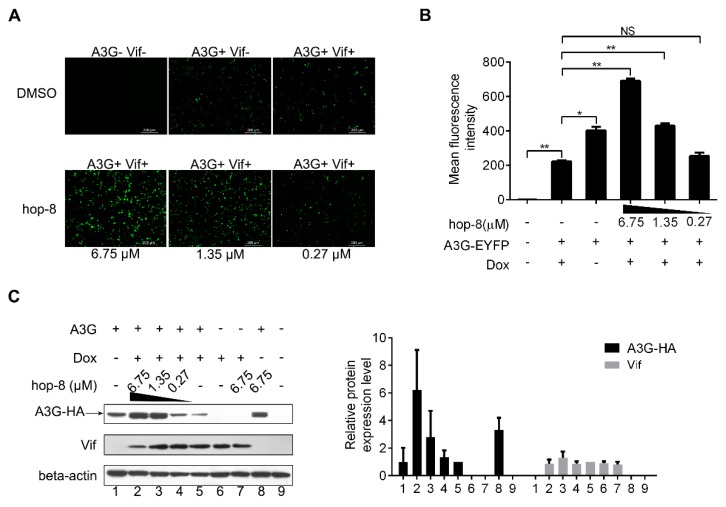
Hop-8 restored A3G levels in cells undergoing Vif-mediated A3G degradation. (**A**) Fluorescence photographs were taken with a fluorescent microscope (Leica DMI4000B); (**B**) Cells were harvested, and EYFP expression in live cells was analyzed with a FACSVerse flow cytometer. Data are expressed as the means ± SD of at least three independent measurements. Statistical comparisons were performed between A3G-EYFP, the Vif positive group (column 2) and other groups with the pair-sample *t*-test with Origin 8.5. (*p* < 0.05, significant difference; *: *p* < 0.05; **: *p* < 0.01; NS: not significant); (**C**) pcDNA3.1-APOBEC3G-HA (0.5 μg) was transfected into TRex-hvif-15 cells. The compound (6.75, 1.35, and 0.27 μM) containing 0.1 μg/mL Dox was added 6 h post-transfection. The relative expression levels of A3G-HA and Vif were normalized by the levels of beta-actin. Values are presented as normalized intensities relative to the values of the Dox and A3G-HA positive groups (lane 5). Each data point represents the mean relative quantity ± standard deviation (bars), n ≥ 3.

To explore the mechanism of antiviral activity of hop-8, a fluorescence-based screening system was used to examine the role of hop-8 in Vif-mediated A3G degradation. Hop-8 was found to recover A3G levels in cells undergoing Vif-mediated A3G degradation. The images showed that with the addition of Dox, A3G expression levels were significantly reduced. The A3G-EYFP (enhanced yellow fluorescent protein) expression was recovered with the addition of hop-8, and this recovery was dose-dependent (Figure 3A). To demonstrate the interference of hop-8 in Vif-mediated A3G degradation, the mean fluorescence intensity (MFI) of EYFP in live cells was analyzed with a FACSVerse^TM^ flow cytometer. The results showed that in the absence of hop-8 and Dox the MFI was 403 ± 21, and 223 ± 7 when Dox was present. The EYFP intensity was picked up significantly with the addition of hop-8 and was dose dependent (Figure 3B).

To elucidate the mechanism underlying the recovery of A3G in cells treated with hop-8, protein expression levels were determined with western blots. The results showed that in cells treated with hop-8, it could upregulate A3G expression and maintain A3G at high levels compared to the cells that were not treated with hop-8. The A3G expression levels were 6.2-, 2.8-, and 1.3-fold higher in cells treated with 6.75, 1.35, and 0.27 μM hop-8, respectively, compared to untreated cells under induced expression of Vif. Meanwhile, the A3G level was significantly higher in hop-8-treated cells with Dox than in cells without Dox, indicating that hop-8 enhanced the A3G expression when Vif was present. (Figure 3C, panel 1, black column chart). Vif expression had no significant changes (Figure 3C, panel 2, gray column chart). These results are consistent with the fluorescence-based primary screen, indicating that hop-8 helped with stand Vif-mediated A3G degradation by keeping A3G at a high level.

### 2.4. Hop-8 Upregulated A3G Expression and its Incorporation in the Progeny Virus Reducing its Infectivity

**Figure 4:**
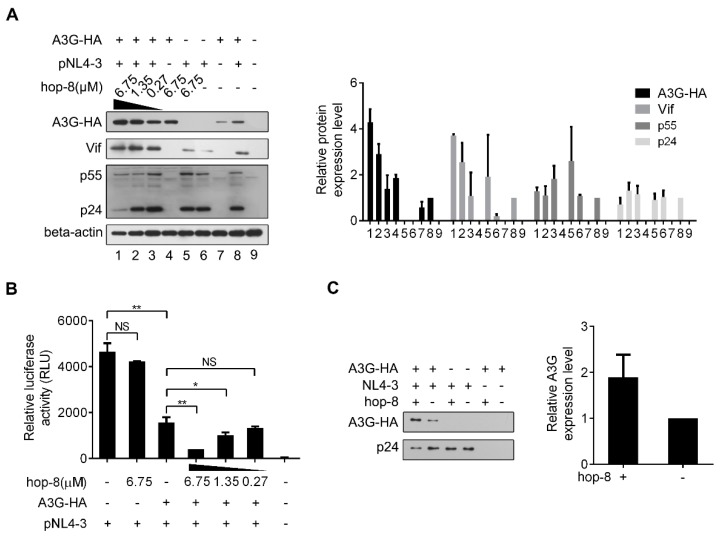
Hop-8 enhanced the expression of A3G and reduced p24 expression in HIV-1 producing cells and reduced the infectivity of the produced virus. (**A**) Hop-8 increased the expression of A3G and Vif while reduced the p24 expression in HIV-1 producing cells. The plasmids pcDNA3.1-APOBEC3G-HA (0.125 μg) and pNL4-3 (0.375 μg) were transfected into 2 × 10^5^ 293T cells. The compound was diluted to 6.75, 1.35, and 0.27 μM with DMEM and added to the wells 6 h post-transfection. The relative expression levels of A3G-HA, Vif, p55, and p24 were normalized by the levels of beta-actin. Values are presented as normalized intensities relative to the values of the pNL4-3 and A3G-HA positive groups (lane 8). Each data point represents the mean relative quantity ± standard deviation (bars), n ≥ 3. (**B**) Hop-8 reduced the infectivity of the progeny virus. The supernatant of NL4-3 was collected and 4 × 10^4^ TZM-bl cells were infected with NL4-3 that contained 20 ng p24. The residual infection was determined using relative luciferase activity. All data represent the means ± standard deviation (bars), n ≥ 3. Data were analyzed by the unpair-sample *t*-test with Origin 8.5. (*p* < 0.05, significant difference; *: *p* < 0.05; **: *p* < 0.01; NS: not significant). (**C**) Hop-8 increased the incorporation of A3G in progeny virions. pcDNA-APOBEC3G-HA (1 μg) and pNL4-3 (1 μg) were transfected consecutively or co-transfected into 293T cells in 6-well cell culture plates. Cells were cultured with or without hop-8 (2.7 μM) for 48 h. The supernatant was collected and centrifuged at 2000× *g* and the cell debris was discarded. The viral particles were lysed with 0.5% Triton-X100. A3G and p24 levels in the supernatant were determined by western blot. The relative expression levels of A3G-HA were normalized by the levels of p24. Each data point represents the mean relative quantity ± standard deviation (bars), n ≥ 3.

To further examine the influence of hop-8 on A3G expression and the infectivity of the progeny virus, pcDNA3.1-APOBEC3G-HA and pNL4-3 were co-transfected into 293T cells. The expression of APOBEC3G-HA, Vif, p55, and beta-actin was analyzed by western blot (Figure 4A). A3G expression in cells treated with 6.75 μM hop-8 was strongly increased compared to untreated cells, and it was dose-dependent. It was interesting that with the transfection of pNL4-3, the cellular A3G level was much higher than that of cells without pNL4-3, suggesting that the effect of hop-8 for increasing the A3G expression was more capable in the presence of HIV infection. (Figure 4A, panel 1). Additionally, Vif expression also increased in hop-8 treated cells (Figure 4A, panel 2). The p55Gag level in cells was increased with the addition of hop-8 in the absence of A3G. While in A3G and NL4-3 co-transfected cells, hop-8 seemed to have little influence in p55Gag expression. The levels of p24 declined in hop-8 treated cells compared with the untreated cells, which were co-transfected with pcDNA3.1-APOBEC3G-HA and pNL4-3. The p24 levels were similar in cells transfected with only pNL4-3 regardless of whether they were treated with hop-8 or left untreated (Figure 4A, panel 3). An infective dose of the supernatant with 20ng of p24 was used to infect TZM-bl cells and luciferase activity was measured to determine infectivity. The results showed that without A3G the virions produced by hop-8 treated cells could infect TZM-bl effectively, and there was no significant difference from the untreated cells. The infectivity of the virus produced with ectopic A3G expression and hop-8 treatment (6.75 μM) decreased by 91% compared with that of the virus from cells with no A3G expression. In A3G expressing cells, the infectivity of the virus produced by hop-8 treatment (6.75 μM) cells was reduced by 70% compared to that of the virus produced by untreated cells. Additionally, the reduction in infectivity of the progeny virus produced by A3G expressing hop-8 treated cells was dose-dependent, indicating that hop-8 probably declined the infectivity of the progeny virus by influencing A3G incorporation (Figure 4B). To check whether hop-8 increased the A3G packaged in progeny virions, the A3G in the supernatant was tested by western blot. The results showed that hop-8 significantly increased the A3G in progeny virions compared with DMSO resulting in the low infectivity of the progeny virus (Figure 4C). These results demonstrated that hop-8 interfered with Vif-mediated A3G degradation by enhancing A3G expression in virus-producing cells, increasing A3G incorporation in progeny virions and reducing the infectivity of the newly produced virus.

### 2.5. Hop-8 Upregulated the Expression of A3G at both the Protein and mRNA Levels

**Figure 5:**
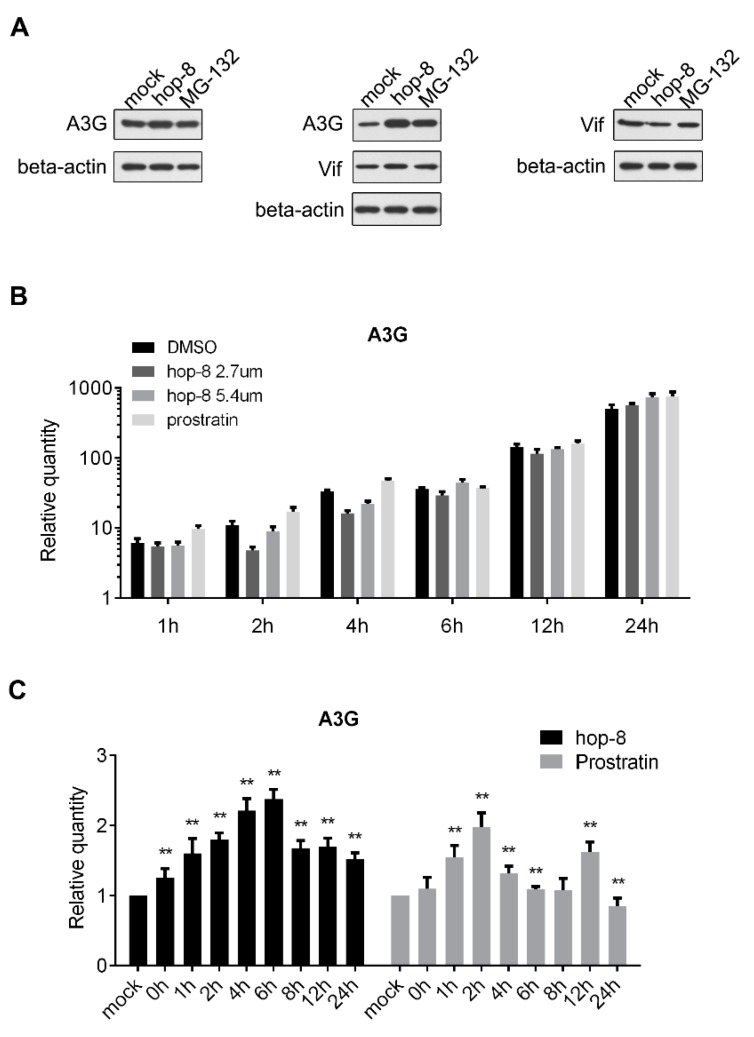
Hop-8 upregulated both the protein and mRNA levels of A3G. (**A**) Hop-8 upregulated A3G protein level regardless of whether Vif was present or absent and had no influence on Vif expression. pcDNA3.1-APOBEC3G-HA and pcDNA3.1-Vif-HA were transfected consecutively or co-transfected into 293T cells and cultured with DMSO, 2.7 μM hop-8, or 10 μM of the proteasome inhibitor MG-132 for 24 h. A3G, Vif, and beta-actin were detected by western blot; (**B**) A3G mRNA levels of pcDNA3.1-APOBEC3G-HA transfected 293T cells in the presence of the 2.7 μM (dark gray) and 5.4 μM (gray) hop-8, prostratin (2.7 μM) (light gray), or DMSO (black). The cells collected at 4 h post transfection (0 h of compounds treatment) were used as a control. Each data point represents the mean relative quantity (relative to the mock) ± standard deviation (bars), n = 3; (**C**) A3G mRNA levels in PBMCs which treated with hop-8 (2 μM) (black) or prostratin (2 μM) (gray). Each data point represents the mean relative quantity (relative to the mock) ± standard deviation (bars), n ≥ 3. Data were analyzed by the Mann Whitney test with GraphPad Prism 6.0 (GraphPad Software, Inc., La Jolla, CA, USA). (*p* < 0.05, significant difference; *: *p* <0.05; **: *p* < 0.01).

To determine the role of hop-8 in upregulating the expression of A3G, pcDNA3.1-APOBEC3G-HA and pcDNA3.1-Vif-HA were transfected consecutively or co-transfected into 293T cells and cultured with DMSO, hop-8, or MG-132. A3G, Vif, and beta-actin were detected by western blot. The results showed that A3G expression was upregulated by hop-8 when Vif was absent. Hop-8, as well as MG-132, raised A3G levels when Vif was present. Besides, hop-8 did not increase Vif expression (Figure 5A). The mRNA level of A3G in cells which were transfected with pcDNA3.1-APOBEC3G-HA was determined by qPCR. The results showed that the cellular A3G mRNA level was higher in hop-8-treated cells than in DMSO-treated cells after 24 h treatment, and the levels were similar in the earlier time. While in prostratin-treated cells the A3G mRNA level was even higher than in hop-8-treated cells (Figure 5B). These results indicated that hop-8 enhanced the A3G gene expression carrying the CMV promoter. The influence of hop-8 on endogenous A3G mRNA levels was measured in PBMCs. The results showed the A3G mRNA levels increased significantly when treated with hop-8 or prostratin and were time dependent (Figure 5C). The A3G mRNA level was first continuously increased and reached the peak at 6 h and then began to decline. A similar phenomenon was observed when cells were treated with prostratin. The A3G mRNA level reached the peak after treatment for 2 h. These results indicated that hop-8 upregulated A3G expression at both the mRNA and protein levels.

### 2.6. Hop-8 did not Interfere in Vif Binding with A3G and Recruiting the Cellular ElonginC/B-Cullin 5 E3 Ubiquitin Ligase Complex

**Figure 6:**
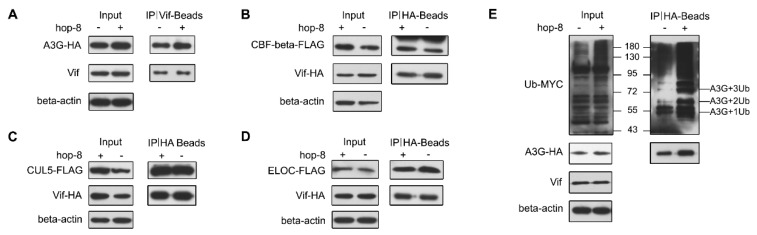
Hop-8 did not interfere in the interaction of Vif with A3G, CBF-β, Elongin C, or Cullin 5. (**A**) Hop-8 did not interfere in Vif and A3G interaction. The plasmid pcDNA3.1-APOBEC3G-HA (3 μg) was transfected into 2 × 10^6^ Trex-hvif-15 cells and treated with 0.1 μg/mL Dox and 2.7 μM hop-8 or DMSO for 48 h. Cells were treated with 10 μM MG-132 for 16 h. Co-IP assays were performed with an anti-HIV1 Vif antibody; (**B**–**D**) Hop-8 did not block the interaction between Vif and CBF-β (**B**), Elongin C (**C**), or Cullin 5 (**D**). The plasmids pcDNA3.1-Vif-HA (1.5 μg) and pcDNA3.1-CBF-β-FLAG (1.5 μg) or pcDNA3.1-Elogin C-FLAG or pcDNA3.1-Cullin 5-FLAG were co-transfected into 2 × 10^6^ 293T cells. Cells were treated with 2.7 μM hop-8 or DMSO. Co-IP assays were performed with an anti-HA antibody; (**E**) The influence of hop-8 on A3G ubiquitination. The plasmids pcDNA3.1-APOBEC3G-HA (1.5 μg) and pUb-MYC (1.5 μg) were co-transfected into 2 × 10^6^ Trex-hvif-15 cells. Cells were treated with 0.1 μg/mL Dox and 2.7 μM hop-8 or DMSO. The Co-IP assay was performed with a rabbit anti-HA antibody.

The interactions between Vif and A3G, CUL5, ELOC, or CBF-β were determined by co-IP assays. The results showed that both in the absence and presence of hop-8, Vif exhibited strong binding with A3G. Hop-8 did not disrupt the interaction between Vif and A3G (Figure 6A). Meanwhile, the co-IP assays between Vif and CUL5, ELOC, or CBF-β demonstrated that hop-8 did not interfere with the interaction of Vif with CUL5, ELOC, or CBF-β (Figure 6B–D). Next, the polyubiquitination of A3G was examined. The results showed that hop-8 did not block A3G ubiquitination (Figure 6E). These results suggest that hop-8 did not inhibit Vif from binding with A3G or CBF-β and recruiting the cellular ElonginC/B-Cullin 5 E3 ubiquitin ligase complex.

## 3. Discussion

In this study, we characterized a novel phorbol ester, 12-*O*-tricosanoylphorbol-20-acetate, that inhibits the HIV spectrum broadly. We first evaluated the antiviral activity of hop-8 against HIV-1 and HIV-2 strains in PBMCs and C8166 cells. Hop-8 inhibited wild-type HIV-1, HIV-2, and drug-resistant strains efficiently. We found that ΔVifNL4-3 showed lower sensitivity to hop-8 and the antiviral activity of hop-8 in PBMCs was better than in C8166. RN-18 and other Vif-A3G inhibitors inhibit HIV-1 efficiently in the nonpermissive PBMCs, and show less activity in the Vif deficient strain [31,33]. The similarity of the antiviral activity against HIV-1 provides circumstantial evidence supporting the fact that the mechanism of hop-8 is associated with Vif and A3G. PBMCs were A3G high-expression cells and C8166 were A3G low-expression cells. The results indicated that one of the possible mechanisms of hop-8 was to protect A3G from Vif degradation. To verify the idea, we used a Vif Tet-on expression system, and found that hop-8 restored A3G levels in cells undergoing Vif-mediated A3G degradation. We demonstrated that hop-8 significantly enhances A3G expression in cells stably expressing Vif by fluorescence imaging, flow cytometry, and western blot assays. Previously identified Vif-A3G inhibitors, such as RN-18, restored the A3G levels in cells but had no ability to raise these levels higher, indicating that hop-8 probably stimulated A3G expression [33]. The treatment of A3G and NL4-3 producing cells showed that hop-8 increased cellular A3G levels and A3G incorporation in progeny virions efficiently. Furthermore, in the presence of A3G, hop-8 reduced the infectivity of the progeny virus. Hop-8 enhanced A3G expression regardless of the presence or absence of Vif in cells. Both the results of the assays measured in TRex-hvif-15 and 293T show that the upregulation of A3G by hop-8 was enhanced in the presence of Vif. The reduced sensitivity of hop-8 against Vif deficient HIV-1 suggests that Vif perhaps play a role in hop-8-mediated A3G protection. A3G could be strongly induced by type I interferon and PMA [36,38]. We found that hop-8 upregulated the mRNA level of A3G in PBMCs and with prostratin, the A3G mRNA level is higher in cells treated with hop-8. The inhibition of HIV-1 by hop-8 in PBMCs was probably by virtue of upregulating A3G expression in cells and helping to protect A3G from Vif-mediated degradation.

The A3G-Vif-CBF-β-CUL5-ELOB-ELOC complex has become a potential target for the design of novel anti-HIV drugs. The first reported Vif small-molecule inhibitor, RN-18, was a Vif–A3G interaction inhibitor that inhibited HIV-1 in nonpermissive cells [33,48]. Several inhibitors targeting the Vif–A3G interaction have been reported in the years following the discovery RN-18, and their mechanisms of action were associated with the A3G-Vif-CBF-β-CUL5-ELOB-ELOC complex [31,35,49,50]. Vif–ELOC interaction is also a target of anti-HIV compounds, and compounds that could inhibit Vif–ELOC interaction have been designed. CUL5 and CBF-β play an important role in Vif-mediated A3G ubiquitination, although no compound has been reported to act as a Vif–CUL5 or Vif–CBF-β interaction inhibitor [51]. These compounds that target the A3G-Vif-CBF-β-CUL5-ELOB-ELOC complex can be regarded as A3G protectors that help maintain normal levels of A3G. To determine whether hop-8 could influence the Vif-mediated ubiquitination ofA3G, the interaction of Vif–A3G, Vif–CBF-β, Vif–CUL5, Vif–ELOC, and A3G ubiquitination were performed. We found that hop-8 had little influence on the interaction between Vif and the other proteins and A3G ubiquitination, indicating that hop-8 had no ability to block Vif-mediated A3G degradation. These results support the idea that the mechanism of hop-8 is completely different from that of the reported Vif-A3G inhibitors.

**Figure 7:**
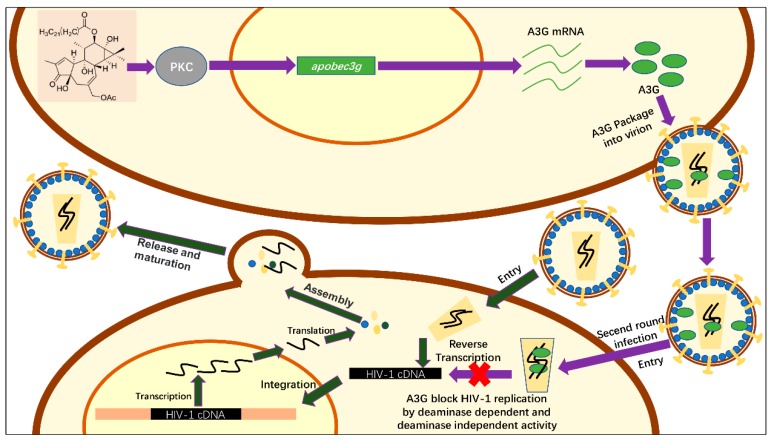
Schematic of the anti-HIV-1 activity mechanism of hop-8. The process shown with deep green arrows represents a simple schematic of HIV replication. Hop-8 stimulates A3G expression via the PKC pathway. A3G is packaged into the progeny virions and restricts the replication of the progeny virions (shown with purple arrows).

The influence of hop-8 on other APOBEC3s showed that apparently hop-8 did not upregulate A3A and A3B (Figure S1A,B). Cells treated with hop-8 had higher A3C, A3D, and A3F than untreated cells (Figure S1C–E). Similar with A3G, the mRNA levels were higher in hop-8 treated cells than in prostratin treated cells. Both hop-8 and prostratin did not increase the expression of A3H (Figure S1F). Besides, prostratin showed a different effect on A3A, A3B, A3C, and A3D. These differences on gene stimulation of hop-8 and prostratin perhaps could be attributable to the molecular structure and size of the two compounds. The high level of A3s also contributed to the antiviral activity of hop-8. Based on the chemical structure of hop-8, we inferred that the possible mechanism of hop-8 stimulation of A3G expression is by activation of the PKC pathway. This inference still needs further studies and elucidation. Besides, we found that hop-8 didn’t stimulate IFN-α, indicating that hop-8 didn’t upregulate A3G via the IFN-α pathway. The over-expression of A3G was measured by transfecting pcDNA3.1-APOBEC3G-HA into cells. Hop-8 upregulated A3G mRNA expression which was turned on by the CMV promotor as well as PMA [52]. However, the enhancing effect was not as significant as in PBMCs. This maybe because the mRNA in A3G overexpression cells was already very high so that the enhancement of the CMV promotor by hop-8 was not significant on the mRNA level, only the protein level. Hop-8 also inhibits the replication of HIV in C8166 cells, which express low levels of A3G. This means that there may exist some other targets of hop-8. The lower sensitivity of NL4-3gp41 (36G) V38A, N42T suggested that hop-8 might target HIV-1 entry. However, no other target of hop-8 was found since the inhibitory activities of reverse transcriptase, proteinase, integrase, and gp41 were determined (Table S1). Earlier studies reported that phorbol esters down-regulated cellular surface CD4, and this down-regulation is mediated by the activation of protein kinase C (PKC) [53,54]. We conjectured that hop-8 probably blocks HIV entry by down-regulating CD4 on the cell surface. But, this still needs further study. We confirm that one of the mechanisms underlying the antiviral activity of hop-8 was stimulating A3G expression to protect against Vif-mediated degradation which helped to resist HIV infection (Figure 7). Inducing A3G expression might play a key role in the antiviral activity of hop-8. As the tumorigenicity of phorbol esters is high, the therapeutic use of hop-8 may be limited. However, the effects of hop-8 on A3G upregulation and HIV inhibition provide a potent strategy for the development of therapeutics for HIV infection.

## 4. Materials and Methods

### 4.1. Ethical Statement

All subjects provided their informed consent for inclusion before they participated in the study. The study was conducted in accordance with the Declaration of Helsinki, and the protocol was approved by the Ethics Committee of Kunming Institute of Zoology, Chinese Academy of Sciences (Approval Number: SWYX-2006011, SMKX-2013016).

### 4.2. Compound

#### 4.2.1. Plant Material

The twigs and leaves of *Ostodes katharinae* were collected from Xishuangbanna, Yunnan Province, P.R. China in August, 2011.

#### 4.2.2. Extraction and Isolation

The dried leaves and twigs of *Ostodes katharinae* (17 kg) were refluxed with 95% methanol for three times. The methanol distillate was concentrated in vacuum to obtain a crude residue. After suspending in water, the crude extract was extracted successively with petroleum ether and ethyl acetate, respectively. The combined these two fractions (270 g) was subjected to silica gel column chromatography, eluted with petroleum ether/ethyl acetate (from 1:0 to 1:1), and finally eluted with MeOH yielding six fractions A–F. Fr. B (25 g) was further separated over a MCI to give seven subfractions (B1–B7), which was purified by Sephadex LH-20 eluted with MeOH and a series of silica gel column chromatography to afford the 12-*O*-tricosanoylphorbol-20-acetate (22 mg) and other analogs with the different fatty acid moieties.

#### 4.2.3. Identification of 12-*O*-Tricosanoylphorbol-20-acetate (hop-8)

*Colourless oil*; ESIMS *m*/*z* 765 [M + Na]^+^; HREIMS *m*/*z* 742.5022 (C45H74O8, calcd. for 742.5784); ${\left[\alpha \right]}_{D}^{16}$ +20.02 (*c* 0.12, CH3Cl); EIMS *m*/*z* 742 (M+), 531 (15), 403 (13), 387 (15), 343 (20), 310 (100), 282 (30). IR (KBr) ν_max_ 3424, 2925, 1748, 1730, 1629, 1377, 1261 cm-1; UV (CH3Cl) λ_max_ 242 nm (11.14); ^1^H-NMR (CDCl3, 400 MHz): 7.59 (1H, s, H-1), 5.70 (1H, d, *J* = 4.5 Hz, H-7), 5.41 (1H, d, *J* = 10.0 Hz, H-12), 4.47 (1H, d, *J* = 12.5 Hz, H-20), 4.42 (1H, d, *J* = 12.5 Hz, H-20), 2.52 (1H, d, *J* = 12.0 Hz, H-5), 2.42 (1H, d, *J* = 12.0 Hz, H-5), 1.05 (1H, d, *J* = 5.1 Hz, H-14), 0.85 (3H, d, *J* = 5.7 Hz, H3-18), 1.75 (3H, s, H3-19), 2.08 (3H, s, 20-OAc); ^13^C-NMR (CDCl3, 100 MHz): 160.8 (C-1), 132.7 (C-2), 208.8 (C-3), 73.4 (C-4), 39.2 (C-5), 135.7 (C-6), 132.7 (C-7), 39.2 (C-8), 78.0 (C-9), 55.9 (C-10), 42.7 (C-11), 76.3 (C-12), 65.4 (C-13), 35.9 (C-14), 25.5 (C-15), 16.7 (C-16), 23.7 (C-17), 14.5 (C-18), 9.2 (C-19), 69.0 (C-20), 173.7, 21.1 (OAc), 173.5 (C-1′), 34.1 (C-2′), 24.8 (C-3′), 28.8-29.5 (C-4′-19′), 31.5 (C-20′), 14.2 (C-21′).

### 4.3. Cells, Virus, and Plasmids

The TREX-hvif-15 (tetracycline inducible expression) cell line, which stably expresses Vif and the A3G-EYFP fusion protein expressing plasmid EYFP-N1-hAPOBEC3G were kindly donated by Prof. Guang-Xia Gao (Institute of Biophysics, CAS). The plasmids pcDNA3.1-Vif-HA, pcDNA3.1-CBF-β-flag, pcDNA3.1-ElonginC-flag, and pcDNA3.1-Cullin5-flag were kindly donated by Prof. Hui Zhang (Institutes of Human Virology, Sun Yat-sen University). The plasmid pUb-MYC was gifted by Prof. Ce-Shi Chen (Kunming Institute of Zoology, CAS). HIV-1_ΔVifNL4-3_ was kindly gifted by Prof. Yong-Hui Zheng (Michigan State University). The plasmids pcDNA3.1-APOBEC3G-HA and pNL4-3, the cell lines 293T and TZM-bl, and the HIV-1 drug-resistant strains HIV-1_A17_, HIV-1_74V_, HIV-1_RF/V82F/184V_, HIV-1_NL4-3gp41 (36G) V38A, N42T_, and HIV-2_CBL-20_ were obtained from NIH AIDS Reagent Program. The human T cell line, C8166, and the laboratory-adapted HIV-1 strain HIV-1_IIIB_ were obtained from Medical Research Council, AIDS Reagent Project and maintained in RPMI 1640 Medium (Life Technologies, Carlsbad, CA, USA) containing 10% fetal bovine serum (FBS, Life Technologies), 100 units/mL penicillin (Sigma, St. Louis, MO, USA) and streptomycin (Amresco, Solon, OH, USA). The cell lines 293T, TZM-bl, and TREX-hvif-15 were maintained in Dulbecco’s modified Eagle medium (DMEM, Life Technologies) containing 10% FBS. PBMCs were isolated from peripheral blood of healthy donors using density-gradient centrifugation with Ficoll standard operating procedure (TBDscience, Tianjin, China) (Ethical Approval Number: SMKX-2013016). PBMCs were stimulated with 5 μg/mL phytohemagglutinin (PHA, Sigma) for 72 h and were cultured in RPMI-1640 containing 10% FBS and 50 units/mL IL-2. HIV-1_IIIB_, HIV-1_A17_, HIV-1_74V_, HIV-1_RF/V82F/184V_, pNL4-3_gp41 (36G) V38A, N42T_, and HIV-2_CBL-20_ were propagated in H9 cells. The clinically isolated strains HIV-1_KM018_ and HIV-1_TC-1_ were isolated from local AIDS patients (Ethical Approval Number: SWYX-2006011) and propagated by co-culture with healthy PBMCs [55,56]. All virus stocks were stored in small aliquots at −70 °C.

### 4.4. Cytotoxicity Assays

The cytotoxicity of the compounds on C8166 and PBMCs were determined by the MTT colorimetric assay as described previously [57]. C8166 cells (4 × 10^4^ cells/well) or PBMCs (5 × 10^5^ cells/well) were co-incubated with serially diluted compounds in 96-well plates at 37 °C with 5% CO_2_. After incubating for 3 days (PBMCs for 7 days), cell viability was determined by using the MTT assay, and the concentration required for 50% cytotoxicity (CC_50_) was determined.

### 4.5. Anti-HIV Activity Assay

C8166 cells were infected with HIV-1_IIIB_, HIV-1_A17_, HIV-1_74V_, HIV-1_RF/V82F/184V_, HIV-1_NL4-3gp41 (36G) V38A, N42T_, or HIV-2_CBL-20_ at different serial dilutions of the compounds with a multiplicity of infection (MOI) of 0.03. PHA-stimulated PBMCs were incubated with HIV-1_KM018_ or HIV-1_TC-1_ in RPMI-1640 (with 10% FBS and 50 U/mL IL-2) at a MOI of 0.1. After incubation at 37 °C for 4 h post-infection, cells were washed three times to remove free viruses and resuspended in RPMI-1640. Next, 100 μl 4 × 10^4^ C8166 cells (5 × 10^5^ cells for PBMC) were seeded in each well of a 96-well plate with a concentration gradient of the compounds to be tested. After incubation for 3 days (7 days for PBMCs), the p24 levels in the culture supernatants (for HIV-2_CBL-20_, the number of syncytia was counted) were determined by using an in-house ELISA assay described previously, and the concentration required for an effectiveness of 50% (EC_50_) was calculated [58,59].

### 4.6. Fluorescence-Based Screening Assay

The Fluorescence-based screening assay was described previously [60]. Briefly, the plasmid EYFP-N1-hAPOBEC3G (0.5 μg) was transfected into TRex-hvif-15 cells (2 × 10^5^ per well) using Lipofectamine 2000 (Life Technology) when the cell confluence was approximately 80%. Doxycycline (Dox) (Clontech, Mountain View, CA, USA) (1 μg/mL) was added to induce Vif expression 6 h post-transfection, also added were serially diluted compounds. Cells were observed with a fluorescent microscope (Leica DMI4000B, Weizlar, Germany) or harvested by trypsin treatment 48 h post-transfection. Dead cells were stained with Fixable Viability Dye eFluor^®^ 660. Enhanced yellow fluorescent protein (EYFP) positive live cells were analyzed. Data acquisition for at least 50,000 events was performed using a FACSVerse^TM^ flow cytometer (BD Biosciences, San Jose, CA, USA), and the data analysis was performed using FlowJo software (Tree Star, Ashland, OR, USA). The plasmid pcDNA3.1-APOBEC3G-HA (0.5 μg) was transfected into TRex-hvif-15 cells (2 × 10^5^ cells/well) using Lipofectamine 2000 when the cell confluence was approximately 80%. Vif expression was induced with 0.1 μg/mL Dox and the compound to be tested was added 6 h post-transfection. After 48 h, the total protein was collected; A3G-HA, Vif, and beta-actin were analyzed by western blot.

### 4.7. HIV-1 Production, Infection, and A3G Incorporation Assay

The plasmids pcDNA3.1-APOBEC3G-HA and pNL4-3 were transfected into 293T cells and cultured with or without hop-8. The cells were collected and the expression of A3G-HA, p55, p24, Vif, and beta-actin was analyzed by western blot. The supernatant was collected and centrifuged at 2000 g and the cell debris discarded. To normalize viral input, the levels of p24 were determined using HIV Type 1 p24 Antigen ELISA (ZeptoMetrix Corporation, Buffalo, NY, USA). TZM-bl cells were infected with supernatant containing 20 ng of HIV-1 p24, and the residual infection was determined using relative luciferase activity. To determine the incorporation of A3G in progeny virion, plasmids pcDNA3.1-APOBEC3G-HA and pNL4-3 were transfected into 293T cells and cultured with or without hop-8 for 48 h. The supernatant was collected and centrifuged at 2000× *g*. The cell debris was discarded. The viral particles were lysed with 0.5% Triton-X100. A3G and p24 levels in the supernatant were determined by western blot.

### 4.8. Real-Time qPCR

The stimulation effect of hop-8 was performed in PBMCs and 293T cells. PHA-stimulated PBMCs were co-cultured with DMSO or hop-8. Total cellular RNA was extracted with RNAiso Plus (Takara, Matsuyama, Japan) after treatment for 0 h, 1 h, 2 h, 4 h, 6 h, 8 h, and 12 h. 0.25 μg pcDNA3.1-APOBEC3G-HA was transfected into 293T cells. 2.7 μM and 5.4 μM hop-8, 2.7 μM prostratin and DMSO were added 4 h post transfection. Cells were lysed with RNAiso Plus when treated for 0 h, 1 h, 2 h, 4 h, 6 h, 12 h, and 24 h. The total cellular RNA was extracted and reverse transcribed into cDNA by using the PrimeScript™ RT Reagent Kit with gDNA Eraser (Perfect Real Time) (Takara). The levels of A3G mRNA were determined by real-time qPCR using a SYBR^®^*Premix Ex Taq*™ II (Tli RNaseH Plus) Kit (Takara) on a 7500 Fast Real-Time PCR System (Life Technologies). GAPDH was used as the endogenous control. The relative levels of A3G mRNA were calculated using the 2^−ΔΔCt^ method [61]. The primers used are shown in Table S2.

### 4.9. Western Blot

Cells were collected and lysed with cell lysis buffer for western blot and IP (Beyotime, Shanghai, China). The total protein was collected and the target proteins were separated by SDS polyacrylamide gel electrophoresis (SDS-PAGE) and transferred to polyvinylidene difluoride (PVDF) membranes (Millipore, Buick Rica, MA, USA). The PVDF membranes with proteins were blocked with 5% milk for 2 h at room temperature and then incubated overnight with primary antibodies at 4 °C. Membranes were probed with horseradish peroxidase (HRP)-conjugated secondary antibodies at room temperature for 1 h. The membranes were washed thoroughly, stained with chemiluminescent HRP Substrate (Millipore), and exposed to X-ray film.

### 4.10. Co-Immunoprecipitation (co-IP)

Forty-eight hours post-transfection, 293T cells were collected and lysed with cell lysis buffer for western blots and IP, and the total cellular protein was collected. Anti-HA or Anti-Vif antibodies were incubated with Protein G Sepharose® beads (Sigma Aldrich) at room temperature for 2 h according to the manufacturer’s instructions. The antibody-conjugated beads were washed with cell lysis buffer four times and incubated with the total cellular protein at 4 °C overnight. Precipitated samples were washed four times, resuspended in PBS, separated by SDS-PAGE, and analyzed by western blot.

### 4.11. Information of the Antibodies

The antibodies used were as follows: anti-HIV1 Vif antibody [319] (ab66643, Abcam); mouse anti-HA antibody [HA-7] (H3663, Sigma-Aldrich); rabbit anti-HA antibody (H6908, Sigma-Aldrich); anti-beta-actin antibody (cw0096a, CWBIO); MYC-tag antibody [2D11A8] (66004-1-Ig, Proteintech Group, Manchester, UK); anti-FLAG M2 antibody (F1804, Sigma-Aldrich); anti-CBFb antibody [EPR6322] (ab133600, Abcam); mouse anti-p24 antibody (produced in-house); anti-mouse IgG (H + L) antibody (4741806, KPL); anti-rabbit IgG (H + L) antibody (0741506, KPL).

### 4.12. Reverse Transcriptase, Protease, Integrase, and gp41 Inhibition Assays

To identify the possible target of hop-8, the inhibition of HIV-1 reverse transcriptase, protease, integrase and gp41 was determined. The inhibition of reverse transcriptase was measured by using Reverse Transcriptase Assay, colorimetric (Roche, Basel, Switzerland) followed the manufacturer’s instructions. The integrase inhibition assay was determined with the HIV-1 Integrase Wild-Type Assay (XpressBio, Frederick, MD, USA) and the protease inhibitory assay was measured by using a SensoLyt™ 490 HIV-1 Protease Assay Kit * Fluorimetric * (ANASPEC). The inhibition of gp41 6-helix bundle formation was determined by ELISA, as described previously [62].

### 4.13. Data Analysis and Statistics

The EC_50_ and CC_50_ values of the inhibitor and other statistical tests were carried out using Origin 8.5 (OriginLab, Northampton, MA, USA) and GraphPad Prism 6.0 (GraphPad Software, Inc., La Jolla, CA, USA).

## 5. Conclusions

We have identified and characterized a novel phorbol ester, 12-*O*-tricosanoylphorbol-20-acetate , which inhibits the replication of clinical isolates in PBMCs and lab-adapted and drug-resistant strains of HIV-1 as well as HIV-2 with different tropisms in C8166 cells. Hop-8 inhibits HIV replication in PBMC mainly by upregulating A3G expression in HIV producing cells and enhancing A3G incorporation in progeny virions. The results of this study might indicate a novel strategy for treating HIV infection.

## Figures and Tables

**Table 1:** The antiviral activity of hop-8.

Cells	Virus	EC_50_ (μM)	
		Hop-8	Prostratin
PBMC	HIV-1_KM018_	0.106 ± 0.003	0.948 ± 0.146
	HIV-1_TC-1_	0.390 ± 0.038	ND ^2^
C8166	HIV-1_IIIB_	0.873 ± 0.005	3.701 ± 0.803
	HIV-1_ΔVif NL4-3_	7.987 ± 0.481	> 10
	HIV-1_NL4-3 gp41 (36G) V38A, N42T_	6.915 ± 1.053	> 10
	HIV-1_A17_	1.303 ± 0.078	3.340 ± 2.075
	HIV-1_RF/V82F/184V_	0.396 ± 0.005	4.069 ± 2.531
	HIV-1_74V_	1.828 ± 0.104	2.457 ± 0.483
	HIV-2_CBL-20_^1^	0.255 ± 0.023	0.568 ± 0.154

^1^ The antiviral activities of the compounds against HIV-2_CBL-20_ were determined by using the cytopathic effect (CPE) assay. ^2^ ND, not determined.

## Supplementary Materials

The following are available online. Figure S1: The influence of hop-8 and prostratin on other APOBEC3s. Table S1: Inhibitive ability of hop-8 against the key enzymes of HIV and gp41, Table S2: Information of the primers used in this study. The spectrums of hop-8 are showed in supplementary.
