# Supplementary material for: Anti-HIV Activities and Mechanism of 12-O-Tricosanoylphorbol-20-acetate, a Novel Phorbol Ester from Ostodes katharinae
Source: Molecules. 2017 Sep 8;22(9):1498. doi: 10.3390/molecules22091498 (PMC6151696; doi:10.3390/molecules22091498)
Supplement: Supplementary file 1 [file molecules-22-01498-s001.zip › hop-8(HREI%2B).pdf]

Elemental Composition Report

Single Mass Analysis

Tolerance = 40.0 mDa / DBE: min = -10.0, max = 120.0
Selected filters: None

Monoisotopic Mass, Odd and Even Electron Ions
30 formula(e) evaluated with 3 results within limits (up to 51 closest results for each mass)
Elements Used:
C: 0-200 H: 0-400 O: 7-9

hop-8 | KIB | Autospec Premier
12:25:38 20-Aug-2012 | M120823EA-03AFAMM 23 (2.111) | P776
Voltage EI+ | 742.5022 | 5.22

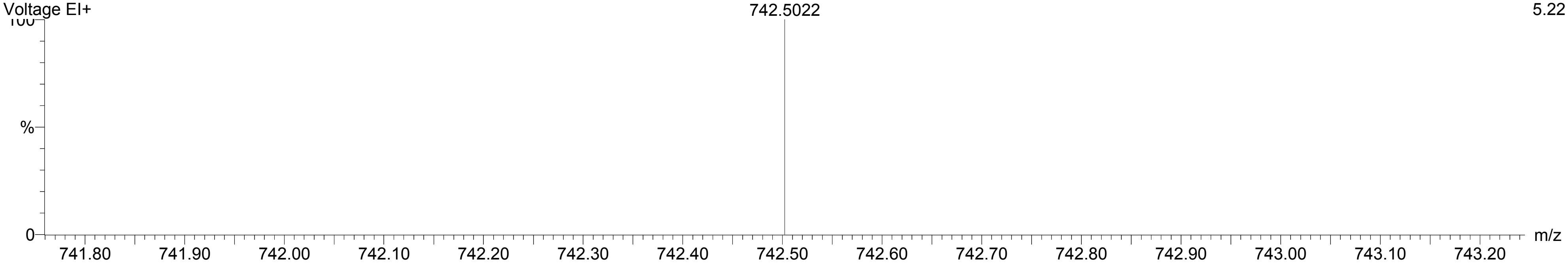

Minimum:				-10.0		
Maximum:		40.0	10.0	120.0		
Mass	Calc. Mass	mDa	PPM	DBE	i-FIT	Formula
742.5022	742.5020	0.2	0.3	10.0	5546025.5	C44 H70 O9
	742.4809	21.3	28.7	15.0	5546026.0	C47 H66 O7
	742.5384	-36.2	-48.8	9.0	5546025.5	C45 H74 O8
