# Supplementary material for: Anti-HIV Activities and Mechanism of 12-O-Tricosanoylphorbol-20-acetate, a Novel Phorbol Ester from Ostodes katharinae
Source: Molecules. 2017 Sep 8;22(9):1498. doi: 10.3390/molecules22091498 (PMC6151696; doi:10.3390/molecules22091498)
Supplement: Supplementary file 1 [file molecules-22-01498-s001.zip › Supplementary materials.pdf]

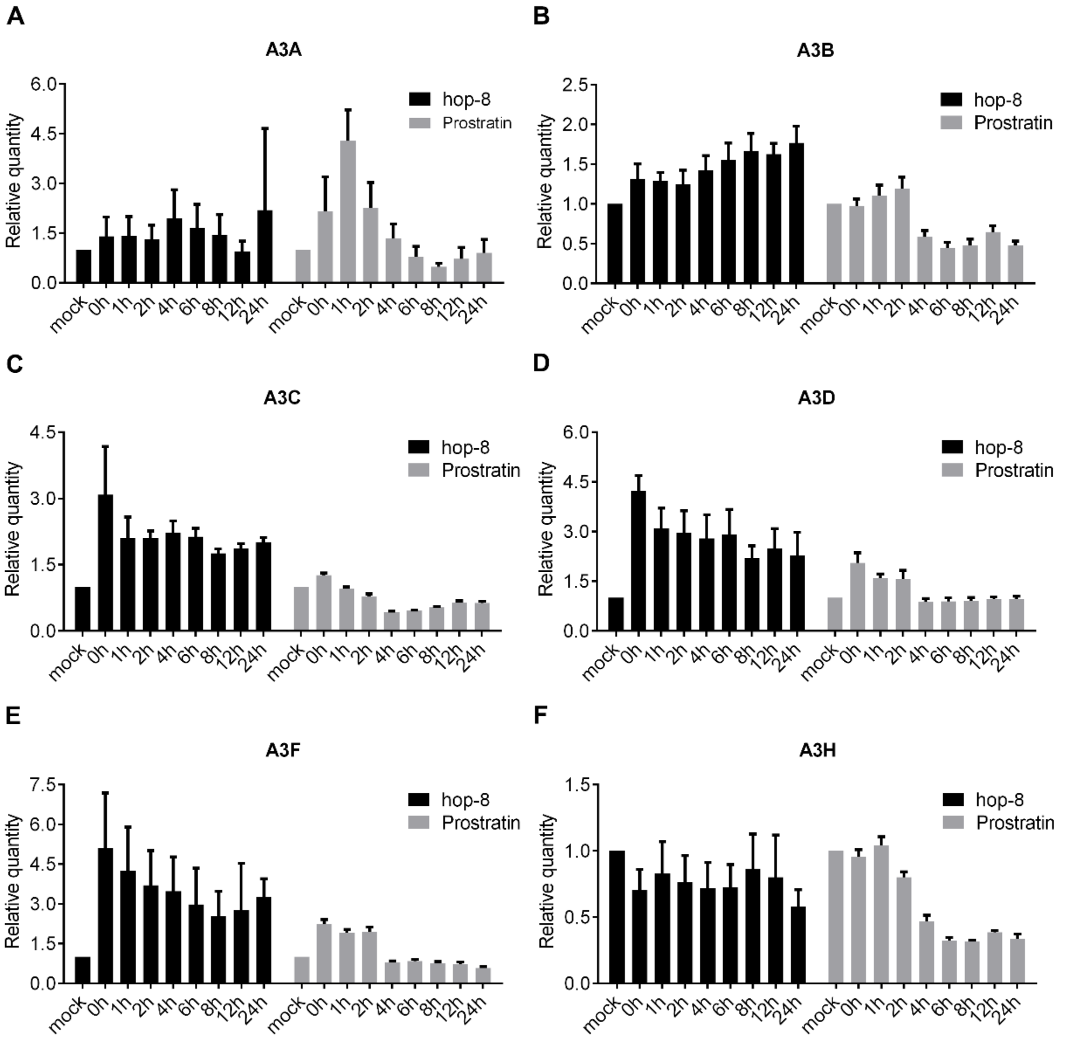

Figure S1. The influence of hop-8 and prostratin on other APOBEC3s. The mRNA levels of A3A (A), A3B (B), A3C (C), A3D (D), A3F (E) and A3H (F) in PBMCs when treated with hop-8 (2μM) (black) or prostratin (2μM) (gray). Each data point represents the mean relative quantity (relative to the mock) ± standard deviation (bars) ± standard deviation (bars), n=2. Data were analyzed with GraphPad Prism 6.0.

Table S1. Inhibitive ability of hop-8 against the key enzymes of HIV and gp41.

Target	IC_{50} (μM)[1]
Reverse transcriptase	>67.5
Protease	>67.5
Integrase	>67.5
gp41	>67.5

[1] IC_{50}, concentration at 50% inhibition, n=2.

Table S2. Information of the primers used in this study.

Gene	GenBank accession No.	Sequence (5′-3′) [1]
GAPDH [2]	M33197.1	5′- GCTTCGCTCTCTGCTCCTCCTGTT-3′ (S)
		5′- ACGACCAAATCCGTTGACTCCGACC-3′ (A)
APOBEC3A	NM_145699.3	5′- CGGGCCCAGACACTTGATGGAT-3′ (A)
		5′- CATTGTCCAGGCGCTCCACTTC-3′ (S)
APOBEC3B	NM_004900.4	5′- ATGGTCGGAGCTACACTTGGCT-3′ (A)
		5′- GGCTTGAAATACACCTGGCCTCG-3′ (S)
APOBEC3C	NM_014508.2	5′- CTGCTCAACTGCAAGGACGCTG-3′ (A)
		5′- ACATTGCCTTCATCGGGTTTCTGA-3′ (S)
APOBEC3D	NM_152426.3	5′- GGGCCGCTCAAATCTCCTTTGG-3′ (A)
		5′- CCGGAAATACACCTCCTGCCTG-3′ (S)
APOBEC3F	NM_145298.5	5′- TTCGAGGCCAGGTGTATTCCCAG-3′ (A)
		5′- ATGGTCAGGGTGACATTGGGGT-3′ (S)
APOBEC3G [2]	JF262036.1	5′- TTGTAGATGAGTGCTCCGTGG-3′ (A)
		5′- CACGTGAGCCTGTGCATCTTC-3′ (S)
APOBEC3H	NM_001166003.2	5′- TGGGCTTCCCAGAGTTTGCTGA-3′ (A)
		5′- CCGTCGCTTTATGGCTCGACTG-3′ (S)

[1] S, sense; A, antisense.

[2] The primers of GAPDH and APOBEC3G were cited from the reference [10].
