# Supplementary figures and images for: Anti-HIV Activities and Mechanism of 12-O-Tricosanoylphorbol-20-acetate, a Novel Phorbol Ester from Ostodes katharinae
Source: Molecules. 2017 Sep 8;22(9):1498. doi: 10.3390/molecules22091498 (PMC6151696; doi:10.3390/molecules22091498)

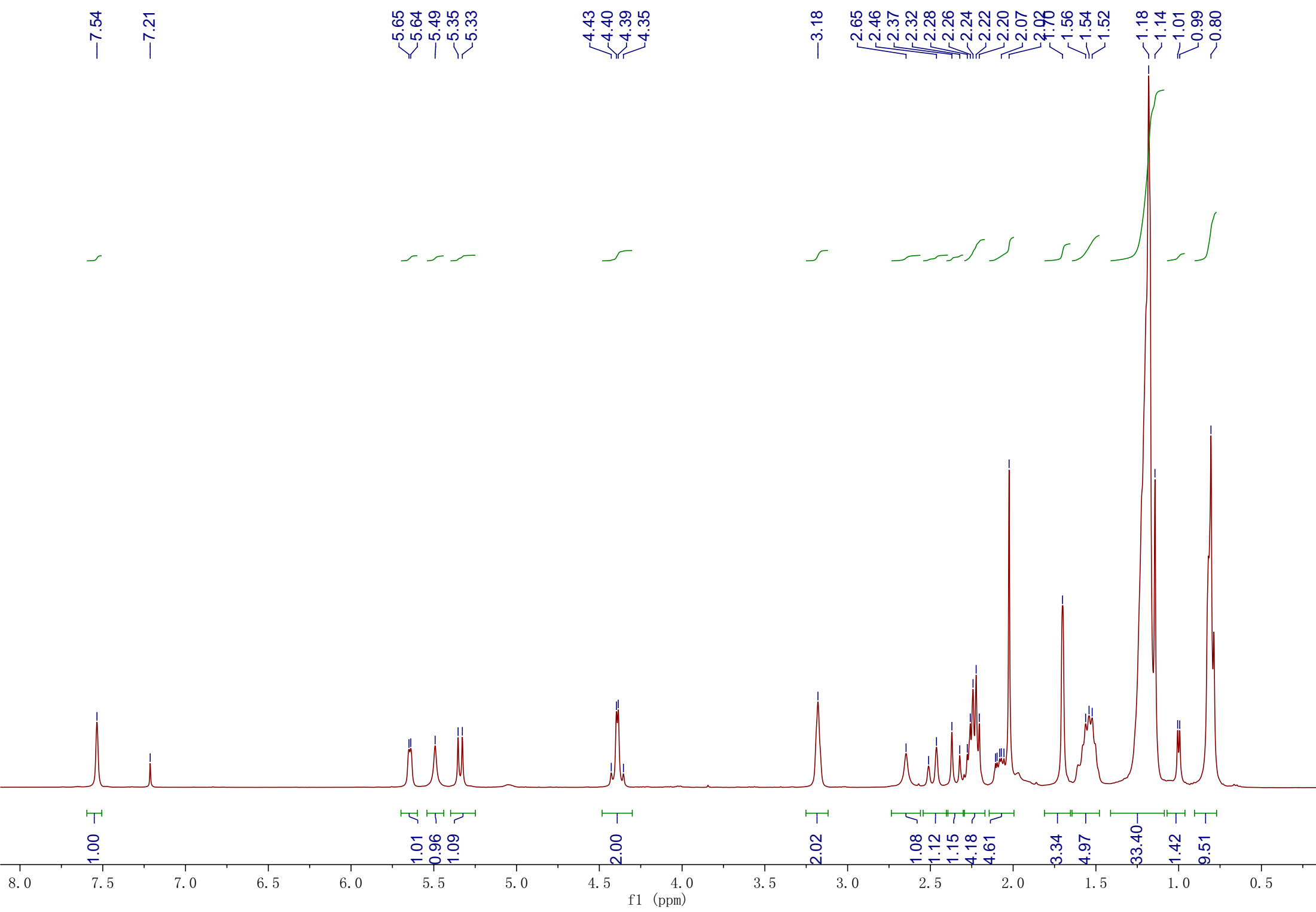

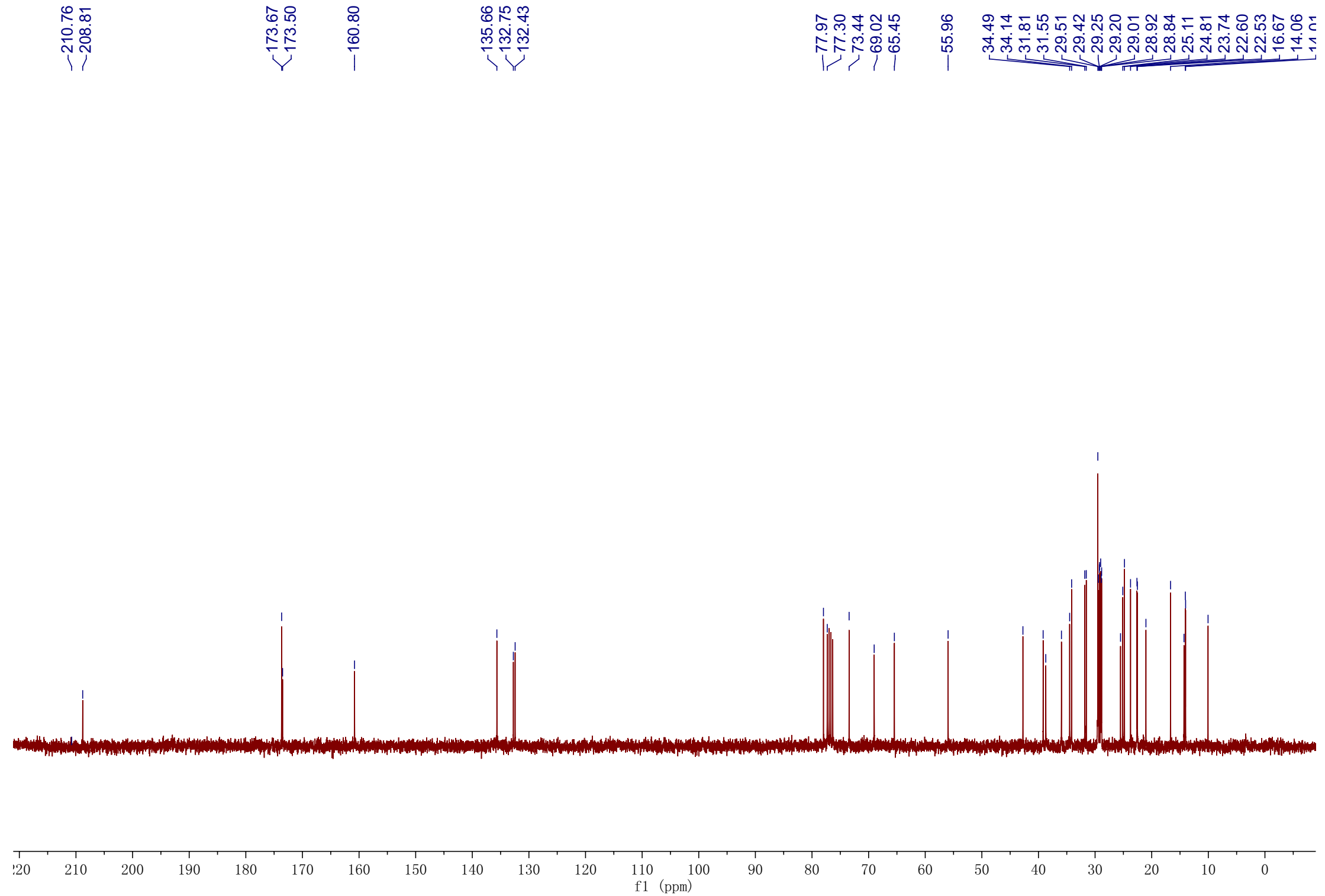

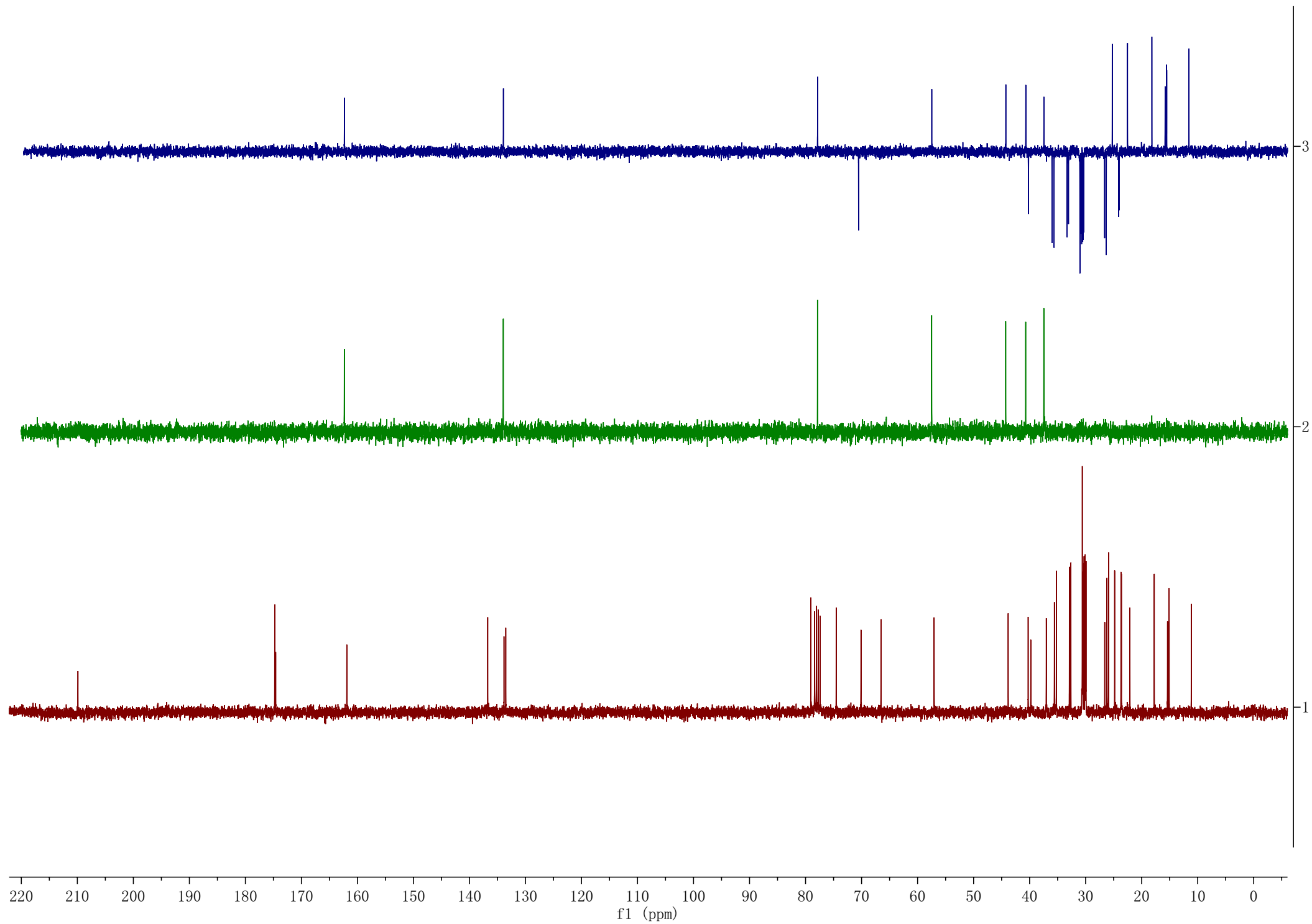

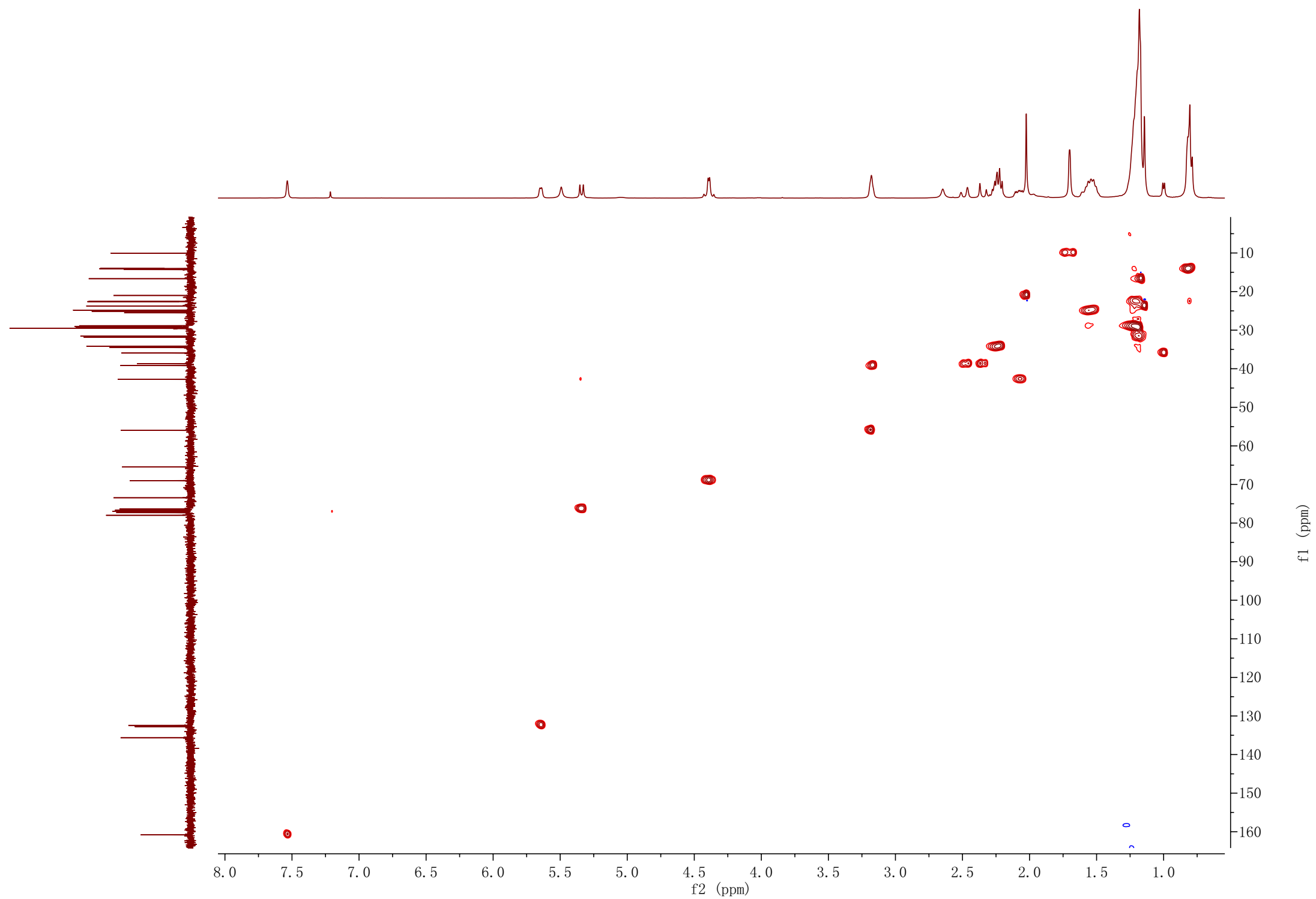

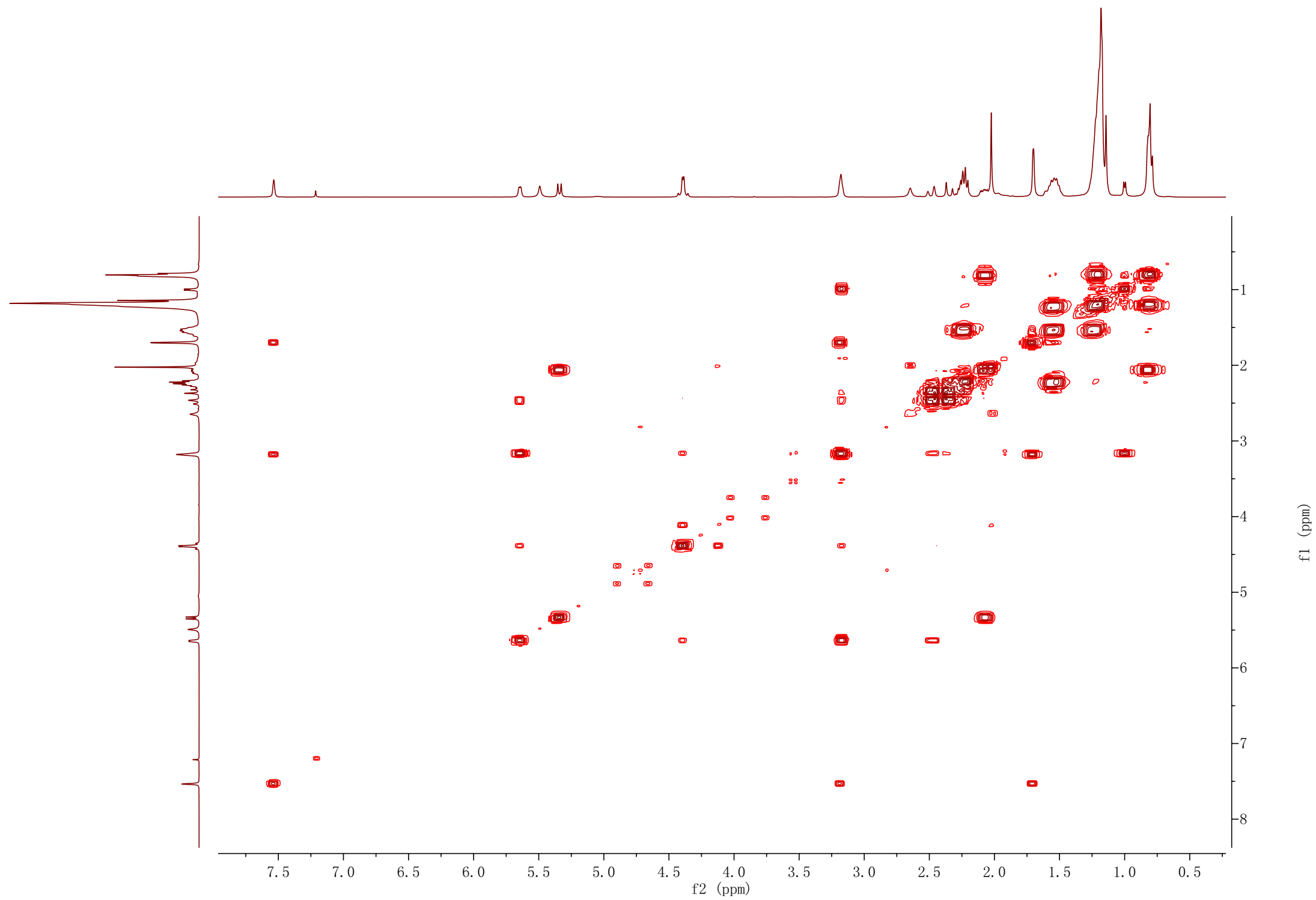

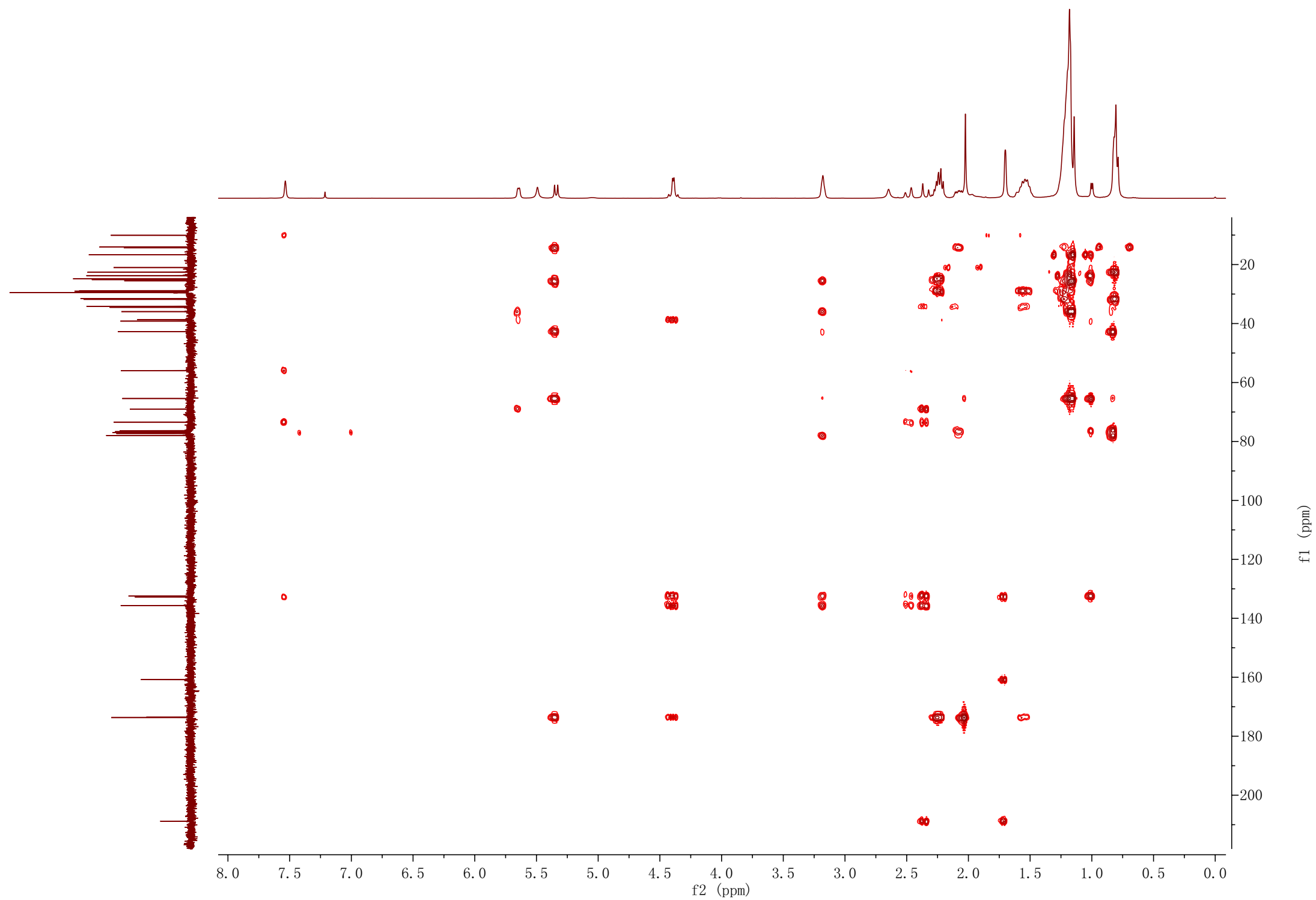

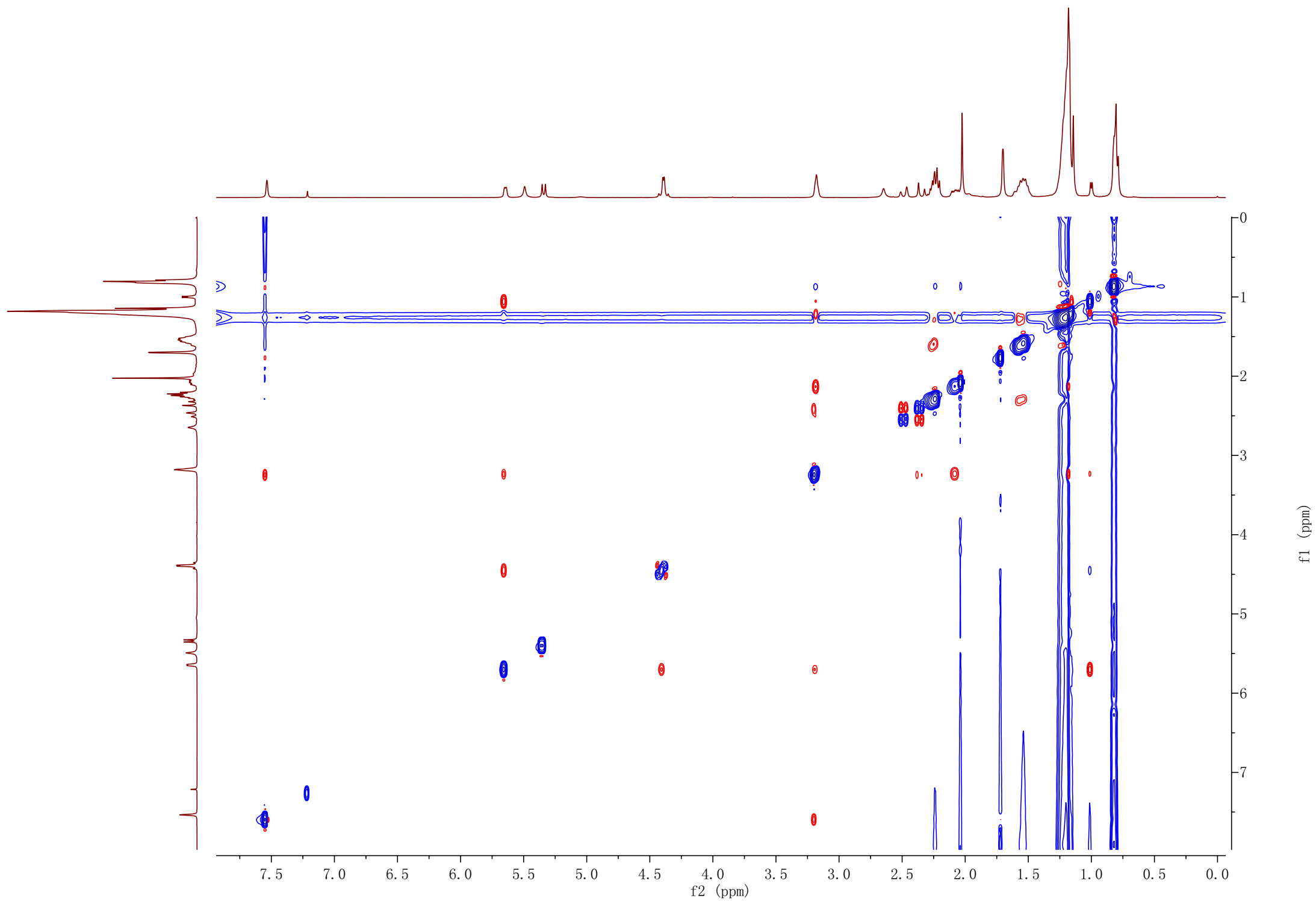

Supplement: Supplementary file 1 [file molecules-22-01498-s001.zip › hop-8(NMR).pdf]

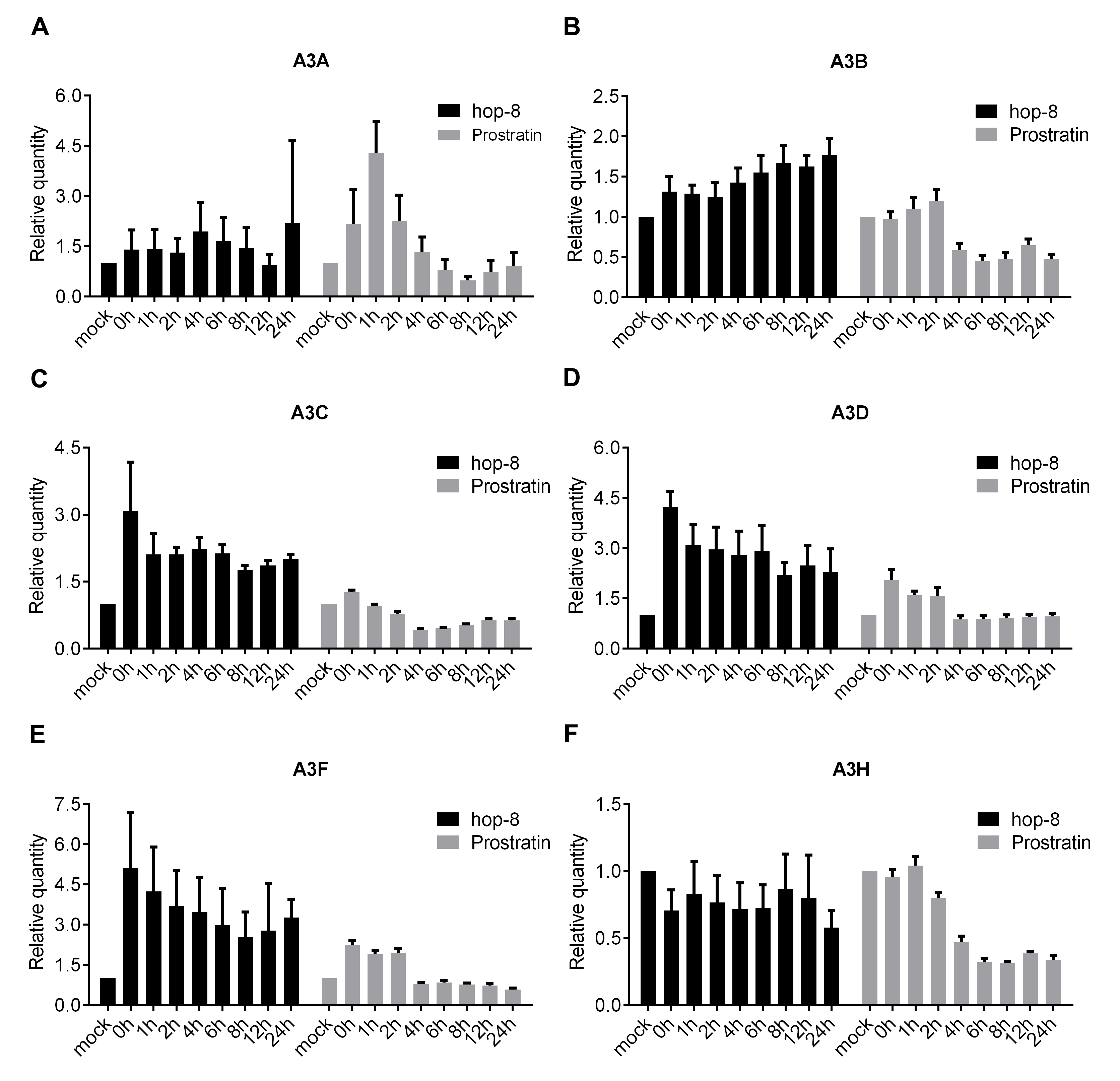

Supplement: Supplementary file 1 [file molecules-22-01498-s001.zip › Figure S1.tif]

hop-8

10:40:46 21-Aug-2017

M170821E-09 27 (1.986) Cn (Top,2, Ht); Cm (21:27-2:14)

KIB

Autospec Premier

P776

Magnet EI+

2.52e3

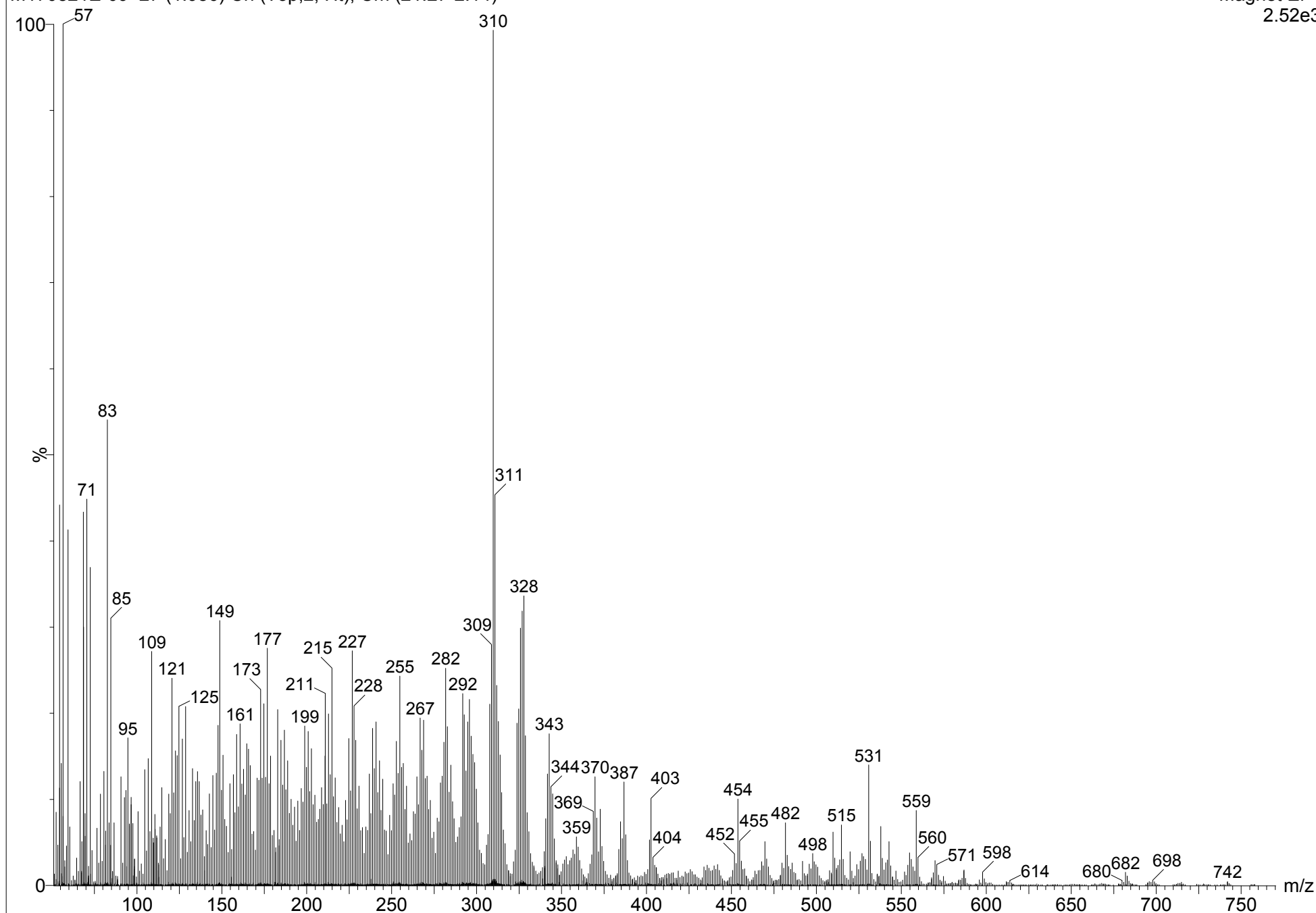

Supplement: Supplementary file 1 [file molecules-22-01498-s001.zip › hop-8(EI+).pdf]
